# Emotion regulation among 4–6 year-old children and its association with their peer relationships in Jordan

**DOI:** 10.3389/fpsyg.2023.1180223

**Published:** 2023-09-08

**Authors:** Amani F. Qashmer

**Affiliations:** Department of Educational Psychology, Faculty of Educational Sciences, The University of Jordan, Amman, Jordan

**Keywords:** emotion regulation, social behavior, Jordan, peer relationship, pro-social, young children

## Abstract

Specific characteristics and competencies are required for maintaining peer relationships, and this study hypothesizes that emotion regulation is one of the competencies. The current study aimed to investigate the association between emotion regulation and peer relationships in 4–6-year-old children, and examine the sex differences among them. This study examined sex differences in peer relationships and the emotion regulation ability of children aged 4–6 years. The study sample comprised 300 children aged 4–6 years [170 girls (56.7%) and 130 boys (43.3%)] studying in kindergarten and first grade in Jordan. As part of data collection, questionnaires were distributed to teachers. The Emotion Regulation Scale (ERC) was used to measure emotion regulation, and the Social Competence and Behavior Evaluation (SCBE) scale, a subscale of the SCBE, was utilized to assess peer relationships. The results revealed a positive relationship between emotion regulation and positive poles of peer relationships (integrated, calm, and pro-social) and a significant negative relationship between emotional lability/negativity and positive poles of peer relationships. These results can be used to design intervention programs to reduce aggressive behavior in children.

## 1. Introduction

Personality, knowledge, skills, habits, and identity are shaped throughout life starting from the early developmental period of childhood (Richter et al., 2015). Children in early childhood exhibit social development and learning potential. They spend a substantial amount of time in kindergarten and schools, which increases their opportunity to establish and build trustworthy, consistent, and secure relationships with their peers (Yuniar, 2021). In early childhood, creating friendships and interacting with peers are normal behaviors. Paulus (2016) elaborated that the predominant feature of this developmental period is making friends, maintaining friendships, participating in a group, and most significantly, developing a sense of others’ needs, which helps develop an altruistic sharing behavior and resource allocation. This in turn helps construct and maintain social relationships with peers.

Peer relationships are interactions between individuals of the same age, characteristics, development level, and with a common lifestyle and social context (Özokçu, 2018). In peer relationships, the age between 4 and 6 years constitutes a period of rapid growth and development in which the children’s ability to get involved and participate in peer groups show the stability of such relationships (Guralnick et al., 2006). The complexity of relationships increases as children transition from preschool to primary school, particularly between 5 and 6 years of age, during which social acceptance and rejection in preschool transform into performing and being involved in more cooperative and collaborative tasks (Andrés-Roqueta et al., 2016). This phase introduces other demands and opportunities for social and emotional development (Gifford-Smith and Brownell, 2003). Transition to primary school implies the transition to a new environment of peers where children become social together, presenting the opportunity to get involved in the second relationship environment that comes after the one of the family (Çiçekoğlu et al., 2019). In primary school, peer relationships become a coping strategy (Walker et al., 2016; McGillicuddy, 2021).

Peer relationships comprise various positive or negative social and psychological behaviors, such as being accepted and liked by peers and being playmates or being unwanted, rejected, and bullied (Çiçekoğlu et al., 2019). The effect of childhood peer relationships on adolescence and later life has been studied extensively in the literature; positive and successful peer relationships in childhood protect against mental disorders in adulthood (Jiang and Wang, 2020; Kehusmaa et al., 2022), whereas children who experience difficulty in peer relationships may develop difficulties in relationships later in their lives (Schwartz et al., 2021).

Observation of children’s behavior with peers involve naturalistic and dominant measurements of peer relationships and children’s behavior. Observational results emphasized that children who were liked by their peers were skilled communicators and leaders, and were social, helpful, sensitive, agreeable, and cooperative (McDonald and Rubin, 2017). Moreover, pro-social behaviors were more likely to result in successful peer relationships and interactions (Wang et al., 2021). In contrast, children who were disliked by their peers were more likely to develop aggressive and disruptive behavior with peers and become shy, withdrawn, socially anxious, and/or experience higher levels of loneliness, particularly in early childhood (LaFreniere and Dumas, 2003; McDonald and Rubin, 2017). While aggression, disruption, and loneliness in children are defined as externalizing problems, those of withdrawal and social anxiety are defined as internalizing problems (LaFreniere and Dumas, 2003; McDonald and Rubin, 2017). Furthermore, these characteristics are the formed as a result of emotional reactions to positive or negative peer behaviors that develop the child’s emotion regulation ability to manage these reactions (Hamaidi et al., 2021).

Emotion regulation is a significant aspect of early childhood. It is a complex management process of emotional arousal (Crowell, 2021). Gülay Ogelman and Fetihi (2021) mentioned emotion regulation as a spontaneous, intrinsic, and environmental process since it involves regulating negative emotions according to the surroundings. Emotion regulation develops during the neonatal period, where regulation is internally and intrinsically organized. In toddlerhood, emotions are not controlled independently, while in the next 2 years, independent control of emotions increases (Crowell, 2021). Emotional development is correlated with social development. Positive and effective interactions between children and their caregivers foster emotion regulation ability (Sroufe, 1996).

Several theories describe the dominant relationship between emotional and social developments. Ekman (1992) proposed that emotions prepare the individual to respond in interpersonal situations. In the preschool period, emotion regulation is developed in the family environment and affected by parents’ behavior and their marital relationship (Chen et al., 2018). Fully independent emotion regulation is related to brain development from 3 to 6 years of age. Children, once exposed to peers, prefer those who exhibit high inhibitory control of negative emotions because children start to integrate negotiation, cooperation, and competitive strategies (López-Pérez and Pacella, 2021).

Hernández et al. (2017) found that children’s emotions are a predictor of their social relationships. Similarly, Gülay and Önder (2013) found that emotions are predictors of pro-social behavior and peer relationships in 5–6-year-old children, and showed that those who express positive emotions exhibit a higher level of pro-social behavior and like their peers more. Similarly, a significant negative correlation has been reported between emotion regulation and internalized and externalized behavior problems. A prevalent finding in peer relationships supports the association between emotion regulation and peer relationships (e.g., Sarac et al., 2020; Gülay Ogelman and Fetihi, 2021).

### 1.1. Emotion regulation in the Jordanian context

In light of the aforementioned discussion, it is evident that it is important to study how children regulate their emotions in international settings. There have been prior studies conducted in Jordan. These studies are significant because they provide insight into emotion regulation among kindergarten students in Jordan within a variety of early education environments. The present study focuses on a variety of aspects of emotion regulation and social competence in Jordanian children. In the Jordanian context, certain research also focused on emotion regulation. One study (Mattar et al., 2020) investigated the relationship between emotion regulation and academic challenges in early childhood education in Jordan. The study looked at the emotion regulation and academic challenges of children in first-grade in Jordan. The findings revealed a significant relationship between emotion regulation and academic difficulties, especially among male students. The results emphasized the importance of emotion regulation in predicting academic achievement.

Alziyoudi and Mattar (2019), in their study, investigated the predictive capacity of cognitive control for emotion regulation and social competence in first-grade students at private schools in Amman, Jordan. The results demonstrated a relationship between cognitive control and emotion regulation in children. Furthermore, the findings revealed no gender differences in the relationship between cognitive control and emotion regulation. Hamaidi et al. (2021) investigated emotion regulation and social competence in kindergarten children. Additionally, the relationship between emotion regulation and social competence, as well as its subdomains (overall emotional adjustment, social interactions with peers, and adults), were examined. The study findings revealed that participants have an average level of emotion regulation and social competence. Children’s emotion regulation and social competence had a statistically significant relationship. While Hamaidi et al. (2021) examined emotional regulation and social competence using all the subdomains, this study focuses on one of the subdomains using a sample comprising participants distributed across a wider age range. In the study of Hamaidi et al. (2021), the participants were exclusively KG2 children. In this study, the participants were KG1, KG2, and first grade students. This research is consistent with recommendations of Hamaidi et al. (2021).

This study employs a correlational descriptive design. The main contribution is to test these variables in the Jordanian context and provide the educators with knowledge about emotion regulation and peer relationship in this period of development.

### 1.2. The present study

A review of previous Jordanian research on emotion regulation revealed that the primary focus was on the relationship between emotion regulation and academic difficulties and school readiness of first grade students (6-year-old). Furthermore, previous research investigated first-graders’ predictive ability of cognitive control for emotion management and social competency with adults and peers. All existing Jordanian studies on emotion regulation recommended further research involving various age groups. As a result, this study focused on kindergarten children’s emotion regulation and its relationship to social competence at the preschool stage.

This study pursued its aim by testing emotion regulation and peer relationship which is an aspect of social competence.

The purpose of this study was to determine the association between peer relationships and emotion regulation levels and examine the differences in both peer relationships and emotion regulation based on sex in 4–6-year-old children. The study hypothesized that peer relationships among 4–6-year-old Jordanian children would be associated with their emotion regulation (e.g., Sarac et al., 2020; Gülay Ogelman and Fetihi, 2021), and that peer relationship and emotion regulation would differ between female and male children (Dunsmore et al., 2008; Chen et al., 2018; Rose and Smith, 2018; López-Pérez and Pacella, 2021). This is the first study in Jordan that investigates emotion regulation and peer relationships and the correlation between them among 4–6-year-old children unlike Hamaidi et al. (2021), who studied social competence with emotion regulation. However, both this study and Hamaidi et al. (2021) focused on the same developmental stage (early childhood), but the participants’ age range is much wider in this study, as described earlier.

## 2. Materials and methods

This study adopted the correlational descriptive method, since it attempted to investigate the association between peer relationships and emotion regulation. Correlational research investigates the existence of a relationship between two variables without addressing the aspects influencing the variables or manipulating them. The study was descriptive because it described the existing relationship between tested variables (Fraenkel et al., 2012).

### 2.1. Participants

Participants were 300 children aged 4–6 years studying at the preschool or elementary school (attending kindergarten and first grade) level in private and public mainstream educational institutions in the university educational province, Amman, Jordan. They had an average age of 5.6 years (standard deviation, SD = 0.54 years). There were 170 girls (56.7%) and 130 boys (43.3%; Table 1). None of the participants had any developmental disorders. They were randomly selected randomly using the simple random sampling from both private and public schools. According to Fraenkel et al. (2012, p.103), the minimum sample size should be 50 for correlational research. Thus, the current sample size was convenient for the study purposes. According to the Ministry of Education, 39,201 students were registered in kindergarten and first grade in Amman for the academic year 2021/2022. The Stephen Thompson equation (380) was utilized to determine the size of the study sample. The author distributed 380 questionnaires; thus, the current sample is deemed convenient and representative of the population. The response rate, however, was (0.79). In the academic year 2021/2022, homeroom teachers completed 300 questionnaires for children after their parents/guardians signed the relevant consent forms.

**Table 1:** Distribution of the sample according to sex.

Independent variable	Category	Number	Frequency/percentage
Sex of children	Male	130	43.3
	Female	170	56.7
Total		300	100

### 2.2. Measures

#### 2.2.1. Emotion regulation

The study used the 24-item Emotion Regulation Checklist (ERC) developed by Shields and Cicchetti (1997). The items are classified into two dimensions: emotion regulation (eight items) and lability/negativity (15 items); the aggregate scores of these two dimensions represent regulation and dysregulation, respectively. Teachers responded to the items on the quartic rating scale from 1 to 4 where 1 = rarely/never, 2 = sometimes, 3 = often, and 4 = almost always. The scale has satisfactory psychometric properties; it has demonstrated a high internal consistency of 0.89 in an American sample (Shields and Cicchetti, 1997). Cronbach’s alpha in a Brazilian sample were 0.84 and 0.74 and 0.96 and 0.83 in a Turkish sample, for lability/negativity and emotion regulation dimensions, respectively (Reis et al., 2016). For the present study, the two dimensions showed a high internal consistency with Cronbach’s alpha of 0.82 and 0.89 for lability/negativity and emotion regulation, respectively.

#### 2.2.2. Peer relationships

The study used the 30-item social interaction with peer’s subscale of the Arabic version of the Social Competence and Behavior Evaluation scale (SCBE), preschool edition (LaFreniere and Dumas, 2003). The scale comprises three subscales related to children’s social interaction with their peers—isolated-integrated (10 items), aggressive-calm (10 items), and egotistical-pro-social (10 items). Each subscale assesses positive and negative qualification poles. Positive qualification poles are integrated, calm, and pro-social, and negative ones are isolated, aggressive, and egotistical. Negative pole items such as “inactive, watches the other children play,” were inversely coded. Teachers responded to the items on a six-point rating scale (1 = almost never to 6 = almost always). The three subscales demonstrated satisfactory psychometric properties in Canadian and American standardization research. The test–retest reliability coefficient in ranged from 0.83 to 0.87 in the Canadian sample and 0.72 to 0.89 in the American sample, and all coefficients were highly significant (*p* < 0.01; LaFreniere and Dumas, 2003). Furthermore, all three subscales had high internal consistency; Cronbach’s alpha ranged from 0.84 to 0.91 in the Canadian sample and 0.81 to 0.87 in the American sample (LaFreniere and Dumas, 2003). In the present study, the subscales showed a high internal consistency, with Cronbach’s alpha ranging from 0.82 to 0.86.

### 2.3. Procedure

Teachers completed both the ERC and SCBE scales for each child. The researcher obtained a signed consent form of participation from either the parents or the legal guardians of the children. Similarly, permissions from the head of the educational department office, university province in Amman, and from school administrations were obtained to conduct the study in both public and private schools. More than 24 teachers volunteered to complete the scales and after receiving the signed consent forms from parents and guardians, they answered the questionnaire during regular working hours. The teachers were engaged with children over 3–9 months, which enabled them to provide specific information about their behaviors. The researcher visited each school and kindergarten at least twice, with the main goal of meeting the teachers and explaining the questionnaire, providing consent forms and to collect the completed questionnaires. To represent the population, 380 questionnaires were distributed. There were 300 completed questionnaires with a response rate of 0.79. The data were collected between November 29, 2021 and May 15, 2022.

### 2.4. Analysis

SPSS 28.0 was used for statistical analyses. The study primarily analyzed coded data using the Pearson correlation test which extracted the Pearson correlation coefficient. The coefficient is a numerical index representing the degree of association between two quantitative variables. Thus, it was appropriate for estimating the association between peer relationship and emotion regulation. Furthermore, the study used the student *t*-test for independently investigating the difference in peer relationship and emotion regulation between girls and boys (Fraenkel et al., 2012).

## 3. Results

### 3.1. Association between peer relationships and emotion regulation

Table 2 shows the correlation between the variables. The variable isolate/integrate correlated negatively with emotional lability/negativity (*r* = −0.314, *p* < 0.01) and positively with emotion regulation (*r* = 0.402, *p* < 0.01); there were positive correlations between aggressive/calm and emotion regulation (*r* = 0.33, *p* < 0.01), egoistic/pro-social and emotion regulation (*r* = 0.26, *p* < 0.01), and emotion regulation and total social interaction with peers score (*r* = 0.398, *p* < 0.01). There were negative correlations between aggressive/calm and emotional lability/negativity (*r* = −0.40, *p* < 0.01), egoistic/pro-social and emotion lability/negativity (*r* = −0.35, *p* < 0.01), and emotion lability/negativity and total social interaction with peers score (*r* = −0.42, *p* < 0.01).

**Table 2:** Correlation among variables.

		Lability/negative	Emotion regulation
Isolated/integrated	Pearson correlation	−0.314^**^	0.402^**^
	Sig. (two-tailed)	0.000	0.00
Aggressive/calm	Pearson correlation	−0.400^**^	0.327^**^
	Sig. (two-tailed)	0.000	0.000
Egoistic/pro-social	Pearson correlation	−0.350^**^	0.258^**^
	Sig. (two-tailed)	0.000	0.000
Social interactions with peers (total)	Pearson correlation	−0.418^**^	0.398^**^
	Sig. (two-tailed)	0.000	0.000

** Correlation is significant at the 0.01 level (two-tailed).

### 3.2. Sex differences in peer relationships and emotion regulation

Mann–Whitney and Wilcoxon tests were performed as non-paramedic tests since the data were not normally distributed. The tests examined the variation in both measures according to sex. Descriptive statistics and Mann–Whitney and Wilcoxon test results for all study variables are shown in Table 3. Results indicated no sex differences in lability/negativity [mean rank for boys (MRboy) = 148.17, sum of ranks (SR) = 19262.0; mean rank for girls (MRgirl) = 152.28, SR = 25888.0, Mann–Whitney = 10747.0, *p* = 0.68], emotion regulation (MRboy = 151.46, SR = 19689.5; MRgirl = 149.77, SR = 25460.5, Mann–Whitney = 10925.5, *p* = 0.87), and social interaction with peers (MRboy = 151.18, SR = 19653.5; MRgirl = 149.98, SR = 25496.5, Mann–Whitney = 10961.5, *p* = 0.91). Similarly, there were no differences in isolated/integrated (MRboy = 150.72, SR = 19593.5; MRgirl = 150.33, SR = 25556.5, Mann–Whitney = 11021.5, *p* = 0.97), aggressive/calm (MRboy = 150.85, SR = 19610.0; MRgirl = 150.24, SR = 25540.0, Mann–Whitney = 11005.0, *p* = 0.95), and egotistic/pro-social subscales (MRboy = 154.74, SR = 20116.0; MRgirl = 147.26, SR = 25034.0, Mann–Whitney = 10499.0, *p* = 0.46).

**Table 3:** The Mann–Whitney and Wilcoxon test results for variables according to sex.

Variables	Gender	*N*	Mean rank	Sum of ranks	Mann–Whitney	Wilcoxon	Sig.
Isolated/integrated	Boy	130	150.72	19593.50	11021.50	25556.50	0.969
	Girl	170	150.33	25556.50			
Aggressive/calm	Boy	130	150.72	19610.00	11005.00	25540.0	0.952
	Girl	170	150.24	25540.00			
Egotistic/pro-social	Boy	130	154.74	20116.00	10499.00	25034.0	0.459
	Girl	170	147.26	25034.00			
Social interactions with peers	Boy	130	151.18	19653.50	10961.50	25496.50	0.905
	Girl	170	149.98	25496.50			
Emotion regulation	Boy	130	151.46	19689.50	10925.50	25460.50	0.867
	Girl	170	149.77	25460.50			
Lability/negativity	Boy	130	148.17	19262.00	10747.00	19262.00	0.684
	Girl	170	152.28	25888.00			

## 4. Discussion

The results of this study revealed a statistically significant association between social interaction with peers and emotion regulation in 4–6-year-old children; positive poles of social interaction with peers (integrated, calm, and pro-social) positively associated with emotion regulation and negatively with emotional lability/negativity.

Children with a higher level of emotion regulation tend to hone their social interactions with peers, presenting higher levels of positive poles. Likewise, a higher level of emotion lability/negativity may lead to more negative poles of social interactions with peers (isolated, aggressive, and egotistic). Children who can adequately express and regulate their emotions exhibit less aggressive and more calm behavior, seek a pro-social solution to conflicts, and do not hurt their peers. Thus, they are favored by their peers. These children also have positive social interaction with peers because they demonstrate high positive abilities to relate to their peers in a caring and considerate manner. Children who regulate their emotions and express their feeling are even-tempered and pay attention to their peers. At the same time, those with a higher level of emotional lability/negativity are aggressive toward their peers, afraid of conflicts and disagreements, reluctant to assert themselves, unable to sustain pro-social change with peers, exhibit little concern for peers’ feelings or perspectives, and are inclined to social conflicts, and are, therefore, less preferred by their peers.

This result accommodates normal behavior in early childhood because children at this age start presenting themselves as social beings and acquiring a spectrum of social competencies, such as expressing their feelings, needs, and opinions, forming social relationships, and assimilating consequences. Hamaidi et al. (2021) stated that interacting with peers is associated with the conscious control of emotions, and that children with higher emotion regulation abilities are more likely to interact with others as they are more capable of managing their negative emotions. Similarly, the ability to successfully interact with peers is more likely to be associated with pro-social behavior (Wang et al., 2021). Children are more likely to prefer peers with high inhibitory control of negative emotion because they can negotiate, cooperate, and compete (López-Pérez and Pacella, 2021). Studies have indicated that peers rejected children who are aggressive and disruptive, shy, withdrawn, socially anxious, and/or have higher levels of loneliness that represent weak emotion regulation ability and higher emotion lability/negativity, which are associated with peer relationship issues (LaFreniere and Dumas, 2003; McDonald and Rubin, 2017; Wang et al., 2021). Other studies found that positive emotions and a high level of emotion regulation are associated with pro-social behavior (Gülay and Önder, 2013) and are preferred by peers (Hernández et al., 2017). The results of this study are consistent with those of prior studies examining the association between emotion regulation and peer relationships (e.g., Sarac et al., 2020; Gülay Ogelman and Fetihi, 2021).

The current study’s findings indicate that there is no significant sex difference in children’s emotion regulation and social interactions with peers. This result contradicts that of studies that reported differences in social interaction with peers (Dunsmore et al., 2008; Rose and Smith, 2018) and emotion regulation (Chen et al., 2018; López-Pérez and Pacella, 2021) between girls and boys. However, consistent with this study, another study on Jordanian kindergarten children (Hamaidi et al., 2021) found no sex difference in emotion regulation and social competence, which included interaction with peers as an aspect of social competence. This inconsistency in sex differences may be due to the social, cultural, and contextual differences between the samples. Moreover, the large sample size of this study may have reduced the differences between girls and boys in social interaction with peers and emotion regulation.

## 5. Conclusion

The study revealed a positive relationship between emotion regulation and positive poles of peer relationships (integrated, calm, and pro-social), and a negative relationship between liability/negativity and positive poles of peer relationships. It is noteworthy that the results are robust to social desirability bias as the teachers responded to the questionnaires. The limitations of the study were its sample size and characteristics; the sample size was relatively small and excluded children with developmental deficits. Future studies should involve a larger sample with diverse characteristics, including children with developmental deficits in both public and private schools. Additionally, data on marital relationship, parenting style, and attachment style were not collected in this study. Future studies should address the potential impact of these variables on peer relationships and emotion regulation. The results of this study recommend addressing the bidirectional prediction model between peer relationships (integrated, calm, and pro-social) and emotion regulation in the same age group. The results can be important for parents of children, educational institutions, and practitioners aiming to develop appropriate prosocial behavior and reduce aggression in children. Furthermore, the findings will aid in the development of training and intervention programs to reduce aggressive behavior among preschoolers. The study’s findings pave the way for future research in this context.

## Data availability statement

The raw data supporting the conclusions of this article will be made available by the authors, without undue reservation.

## Ethics statement

The studies involving humans were approved by The Ministry of Education in Amman, Jordan. The studies were conducted in accordance with the local legislation and institutional requirements. Written informed consent for participation in this study was provided by the participants’ legal guardians/next of kin.

## Author contributions

AQ: study conception and design, data collection, analysis and interpretation of results, and manuscript preparation.

## Conflict of interest

The author declares that the research was conducted in the absence of any commercial or financial relationships that could be construed as a potential conflict of interest.

## Publisher’s note

All claims expressed in this article are solely those of the authors and do not necessarily represent those of their affiliated organizations, or those of the publisher, the editors and the reviewers. Any product that may be evaluated in this article, or claim that may be made by its manufacturer, is not guaranteed or endorsed by the publisher.
